# Rapid Detection of Methicillin-Resistant *Staphylococcus aureus* Directly from Blood for the Diagnosis of Bloodstream Infections: A Mini-Review

**DOI:** 10.3390/diagnostics10100830

**Published:** 2020-10-15

**Authors:** Anna Rita Buonomini, Elisabetta Riva, Giovanni Di Bonaventura, Giovanni Gherardi

**Affiliations:** 1 Department of Medicine, Campus Biomedico University, Via Alvaro del Portillo 200, 00128 Rome, Italy; a.buonomini@unicampus.it (A.R.B.); e.riva@unicampus.it (E.R.); 2 Department of Medical, Oral and Biotechnological Sciences, G. d’Annunzio” University of Chieti-Pescara, Via Vestini 31, 66100 Chieti, Italy; g.dibonaventura@unich.it; 3 Center for Advanced Studies and Technology (CAST), “G. d’Annunzio” University of Chieti-Pescara, Via Luigi Polacchi 11, 66100 Chieti, Italy

**Keywords:** *Staphylococcus aureus*, methicillin resistance, bloodstream infections, rapid tests

## Abstract

*Staphylococcus aureus* represents a major human pathogen able to cause a number of infections, especially bloodstream infections (BSI). Clinical use of methicillin has led to the emergence of methicillin-resistant *S. aureus* (MRSA) and MRSA-BSI have been reported to be associated with high morbidity and mortality. Clinical diagnosis of BSI is based on the results from blood culture that, although considered the gold standard method, is time-consuming. For this reason, rapid diagnostic tests to identify the presence of methicillin-susceptible *S. aureus* (MSSA) and MRSA isolates directly in blood cultures are being used with increasing frequency to rapidly commence targeted antimicrobial therapy, also in the light of antimicrobial stewardship efforts. Here, we review and report the most common rapid non-molecular and molecular methods currently available to detect the presence of MRSA directly from blood.

## 1. Introduction

*Staphylococcus aureus*, a Gram-positive coagulase-positive pathogen belonging to the family *Staphylococcaceae*, usually acts as a commensal of the human microbiota but it can also become an opportunistic pathogen due to the ability to adapt to different host and environmental conditions and to cause many infections [1,2,3,4]. It can cause skin, soft tissue, and lower respiratory tract infections, as well as those related to medical instrumentation, and some serious deep-seated infections such as osteomyelitis and endocarditis. Indeed, *S. aureus* continue to be leading cause of bloodstream infections (BSI) [5].

A major clinical concern for *S. aureus* is its ability to acquire antibiotic resistance, particularly methicillin resistance consequent to methicillin usage, and methicillin-resistant *S. aureus* (MRSA) has been associated with high morbidity and mortality rates [6,7,8]. MRSA strains carry an altered penicillin-binding protein (PBP 2a or PBP 2′) with decreased binding affinity for penicillin encoded by the gene *mecA* carried on a mobile genetic element, the staphylococcal cassette chromosome *mec* (SCC*mec*) [9,10,11,12,13]. A novel *mecA* homologue determinant, named *mecC*, has been rarely identified among MRSA [14]. MRSA isolates are, therefore, resistant to most available beta-lactam drugs, whilst they remain susceptible to the new cephalosporins ceftaroline and ceftobiprole.

*S. aureus* BSI are associated with a 30-day mortality up to 46% [15,16,17]. Earlier appropriation of antibiotics has been associated with reduced mortality in the setting of BSI, including *S. aureus* bacteremia [18,19,20,21]. Clinical diagnosis of BSI depends on traditional blood culture results, although bacterial identification by these conventional methods is time-consuming. Molecular diagnostic tests to identify the presence of methicillin-susceptible *S. aureus* (MSSA) and MRSA isolates directly in critical clinical specimens, such as blood cultures, are being used, providing results in 1–5 h without the requirement for bacterial growth onto agar media, with increasing frequency in guiding antimicrobial therapy for staphylococcal infections as part of antimicrobial stewardship programs [22,23,24,25,26,27]. In recent years, several molecular panels able to simultaneously detect many organisms, including MSSA/MRSA, in one sample have been developed for the diagnosis of infectious syndromes. Depending on the test used, MRSA can be detected 12–48 h earlier than with traditional methodologies [28,29,30].

In this paper, we provide an overview of the most commonly reported non-molecular and molecular rapid methods currently available to detect the presence of *S. aureus* and MRSA directly from blood. Table 1 summarizes the commercially available blood culture-independent and -dependent methods.

## 2. Molecular Blood Culture-Independent Methods for Identification Directly from Blood Samples

In these methods (Table 1), the identification of *S. aureus* directly from blood samples can take place during or after nucleic acid amplification by multiplex real-time PCR [31,32]. The automated system LightCycler SeptiFast (Roche Molecular System, Basel, Switzerland) is able to identify 19 pathogens including *S. aureus* and *mecA*, and in the multistep automated system MagicPlex Sepsis Test (Seegene, Seoul, Korea) conventional and real-time PCR are associated to detect several pathogens included *S. aureus* and some resistance genes including *mecA* [31]. Identification systems directly from blood samples after amplification are carried out in the SepsiTest (Molzym, Bremen, Germany), a semiautomated system based on broad-range PCRs using universal primers, followed by sequencing by BLAST with a time to result ranging between 8 and 12 h; the VYOO system (SIRS-Lab, Jena, Germany) DNA amplification method based on multiple PCRs that target the 16S rRNA gene followed by an electrophoresis on an agarose gel with a time to results of about 8 h; and the PLEX-ID Iridica (Abbott Molecular, Abbott Park, IL, USA), a universal molecular device that can detect and identify up to 800 pathogens, including *S. aureus*, coupling microbe detection by PCR and amplicon analysis by electrospray/ionization mass spectrometry (ESI-MS) with a time to result of about 6–8 h [31,33]. Recently, the automated T2Dx instrument platform (T2 Biosystems, Lexington, MD, USA) has been developed using T2 magnetic resonance (T2MR) technology to provide a ‘sample-to-result’ clinical diagnostic test with final results obtained by 6 h. This enables multiplex detection of ESKAPEc (*Enterococcus faecium*, *S. aureus*, *Klebsiella pneumoniae*, *Acinetobacter baumannii*, *Pseudomonas aeruginosa*, *Escherichia coli*) pathogens in a single whole-blood sample, achieving overall sensitivity and specificity rates of 90 and 98%, respectively [34,35].

## 3. Positive Blood Culture-Dependent Methods for Identification and Antimicrobial Susceptibility Testing of *S. aureus*

Blood culture-dependent methods for diagnosis of *S. aureus* BSI can be divided into systems applied to different protocols: (i) directly from positive blood culture bottles; (ii) directly from positive blood cultures from pellets; and (iii) from early subcultures after a short incubation period of approximately 3–4 h [32]. For the latter two protocols that use pellets and early subcultures, identification and antimicrobial susceptibility testing require a pure subculture, and MALDI-TOF MS, currently considered the best method for rapid bacterial identification, with the two most common platforms represented by Microflex (Bruker Daltonics, Billerica, MD, USA) and Vitek MS (bioMérieux, Marcy l’Etoile, France), is used for bacterial identification [36,37,38,39]. Identification using MALDI-TOF MS is based on the comparison of surface and ribosomal protein profiles obtained by mass spectrometry with a database of protein profiles of well characterized bacterial species [38].

For the protocol applied directly to positive blood culture bottles, the major molecular methods are fluorescence in situ hybridization (FISH), microarrays and some rapid PCR-based tests [38]. FISH is a fluorescence microscopy method based on the specific binding of fluorescent nucleic acid probes on complementary bacterial 16S rRNA sequences. The panel available for staphylococci is the *S. aureus*/coagulase-negative [38]. The commercial solutions PNA-FISH and Quick-FISH (AdvanDx, Woburn, MA, USA) display a time to result of about 1.5 to 3 h [38]. Recently, a rapid and direct detection of *S. aureus* in 41 positive blood cultures through three molecular beacon-based probes by fluorescence in situ hybridization (MB-FISH) has been described, with specificity and sensitivity of 100% and 93.75%, respectively [40].

Microarrays generally contain probes for the detection of the most common pathogens along with resistance genes. The Verigene system (Nanosphere, Northbrook, IL, USA) comprises distinct kits for Gram-negative and Gram-positive bacteria and allows the detection of 12 Gram-positive included *S. aureus* [41,42]. In a study evaluating the performance of the Verigene Gram-positive blood culture assay (BC-GP, Luminex Corporation, Austin, TX, USA), all MRSA tested were correctly identified [43]. The Prove-it Sepsis assay (Mobidiag, Esbo, Finland) is another molecular microarray which combines broad-range PCR and microarray-based assay with a turnaround time of 3.5 h [44].

Rapid multiplex real-time PCR assays that allow both detection and identification of MSSA and MRSA have been developed for blood cultures, such as GeneXpert MRSA/SA BC Assay (Cepheid, Sunnyvale, CA, USA), and the BD Max StaphSR assay (Becton Dickinson GeneOhm, San Diego, CA, USA), with results available in approximately 1.5 h [45,46,47,48]. The GeneXpert MRSA/SA BC 2013 test detects three targets: the gene encoding staphylococcal protein A (*spa*), *mecA*, and the junction region between *orfX* chromosome and the SCC*mec* element. In the updated version, rule-based algorithms allow differentiation between MSSA and MRSA; further, it has successfully detected both *spa* and SCC*mec* variants of MRSA, although genetic variations that may interfere with Xpert MRSA/SA BC test results have been observed, if rarely [49]. The BD Max StaphSR assay is an automated qualitative in vitro diagnostic test using a simplified molecular platform with minimal hands-on time; it requires minimal technical skill with results available within 2 h of the blood culture signaling positive, with excellent sensitivity (MRSA: 100%; MSSA: 99.1%) and a specificity of 100% [50]. A comparison of the next-generation Xpert MRSA/SA BC test and the GeneOhm StaphSR assay for identification of MSSA/MRSA in positive blood cultures reported a sensitivity of 99.2% and a specificity of 96.5% for identifying *S. aureus*, as well as a sensitivity of 94.3% and a specificity of 97.8% for identifying MRSA [51].

Among PCR-based methods, the FilmArray BCID panel (Idaho Technology, Salt Lake City, UT, USA) also needs to be mentioned due to its common use. It is a multiplex PCR-based system designed to detect in positive blood cultures the most prevalent pathogens and resistance genes, including *S. aureus* and *mecA* [52]. Moreover, loop-mediated isothermal amplification (LAMP), an amplification technique that uses Bst DNA polymerase with strand displacing activity, has also been introduced to rapidly identify the most common pathogens and their major resistance genes from positive blood culture bottles within a time frame of 30 min [53,54,55]. Among three different eazyplex^®^ LAMP assays available, the eazyplex^®^ MRSA (Amplex BioSystems, Giessen, Germany) is a CE-labeled commercial test for the detection of *S. aureus*, *S. epidermidis*, *mecA*, and *mecC* from nasal and pharyngeal swabs, but it has also been used with a preliminary blood culture protocol, with sensitivity and specificity of almost 100% [56]. A novel and unique approach is the Accelerate Pheno system (Accelerate Diagnostics, Tucson, AZ, USA) that allows identification of several bacteria included *S. aureus* and fungi by FISH, as well as phenotypic antimicrobial susceptibility testing by morpho-kinetic cellular analysis directly from positive blood culture [57].

A few rapid non-molecular methods have also been developed for rapid identification of MRSA from blood culture. The Direct Tube Coagulase Test has been described as a rapid, simple, and inexpensive method [58]. After confirmation by Gram staining, clot formation is assessed after incubation of a mixture of drops of culture broth and 0.5 mL of rabbit plasma at 35 °C for 2 and 4 h, with sensitivity and specificity of about 80% and 100%, respectively [58,59]. Indeed, the rapid BinaxNOW *Staphylococcus aureus* (BNSA) immunochromatographic test (Alere Scarborough, Inc., Scarborough, ME, USA) was found to accurately differentiate *S. aureus* from coagulase-negative staphylococci (CoNS) and other Gram-positive cocci directly from blood culture bottles, within 30 min from the time of blood culture positivity with sensitivity and specificity of 95.8% and 99.6%, respectively [60].

## 4. Other Rapid Antimicrobial Susceptibility Testing (AST) Phenotypic Methods Directly on Positive Blood Cultures

For rapid AST, besides the methods cited above, other phenotypic approaches have been described with or without a subculture step (Table 2). They can be performed directly from positive blood culture bottles, by bacterial pellet or after an early incubation protocol. The Accelerate Pheno system has been previously cited and allows phenotypic antimicrobial susceptibility testing by morpho-kinetic cellular analysis with time to result of approximately 7 h. Other rapid AST phenotypic methods include the automated broth microdilution method by Phoenix and Vitek, disk diffusion methods, and gradient strips using Etest (bioMérieux) or MIC Strip (Liofilchem) [61,62,63]. Rapid AST can be also performed directly on blood culture samples tested on Mueller–Hinton Rapid agar (MHR-SIR) with a time delay of 6–8 h associated with a significant time saving (17 h), according to the appropriateness of the antibiotic prescription [64,65]. AST by Alfred AST system performed directly from positive blood bottles has also been recently reported. This system, based on the detection of growth by turbidimetry through a technology based on light scattering, is considered a valid and fast method to obtain antimicrobial susceptibility results within the same work shift after blood culture positivity [66]. Semi-quantitative MALDI-TOF for growth rate analysis has been described as a useful method to provide a resistance profile independent of resistance mechanism for antimicrobial susceptibility testing in *S. aureus*, yielding an overall accuracy rate of 95%, including oxacillin susceptibility testing [67]. It is also worthwhile to mention specific protocols that offer a significant benefit to early detection of MRSA in positive blood cultures. For example, the blood culture-dependent three-hour short incubation protocol combines MALDI TOF identification with the qualitative immunochromatographic PBP2a detection test assay (Alere) to detect methicillin resistance, yielding a sensitivity and specificity of 87.5% and 100%, respectively [68,69]. Recently, a revised version of an immunochromatographic assay for detection of *mecA*- and *mecC*-positive MRSA has been described [70].

## 5. Conclusions

In this mini-review, we have reported and discussed the main rapid methods currently available to detect the presence of MSSA/MRSA directly from blood. The major limitation of this paper is due to the difficulty in making a systematic review from the literature due to the scarce reports that could be retrieved among the published papers using specific and targeted keywords. Therefore, some previously published reviews have been retrieved that describe rapid diagnostic methods not specifically used for MRSA bacteremia but applied to all possible pathogens causing BSI.

Multidrug-resistant Gram-negative bacteria, such as carbapenem-resistant *P. aeruginosa* and *A. baumannii*, as well as carbapenem-resistant and extended spectrum beta lactamases-producing *Enterobacterales*, represent the highest priority of resistant pathogens for which new antibiotics are urgently needed, as indicated by the World Health Organization [71]. Treating severe infections due to multidrug-resistant Gram-negative bacteria is one of the most important challenges for clinicians worldwide and the major efforts of the scientific community have been mainly focused on the development of rapid microbiological tests for BSI due to multidrug resistant Gram-negative bacteria [72]. However, it has been reported that in routine patient care *S. aureus* is the most common pathogen isolated from blood and, together with *Enterobacterales*, accounts for approximately half of all blood culture isolates in most studies [30]. Optimal patient care requires access to necessary laboratory testing including clinical microbiology, and a rethinking of hours of operation is required, i.e., 24 h/7 days a week open laboratory, to shorten time of accurate result reporting [73]. Rapid identification and differentiation of *S. aureus* from CoNS in blood culture bottles is reported to have a major impact on improving patient outcomes, decreasing length of hospital stays, and reducing health care expenses [23,74,75]. Inappropriate antibiotic therapy in cases of *S. aureus* BSI has been found to be associated with a higher risk of mortality and extended hospital length of stays, therefore appropriate antimicrobial therapy seems to be the most important factor associated with a better clinical outcome. Even though MRSA epidemiology varies between countries, MRSA risk factors can be easily identifiable, such as previous MRSA infection or colonization, coming from long-term care facility, and chronic renal diseases [76]. The risk factors should be also considered before expensive rapid diagnostic tests are performed. Conventional identification of *S. aureus* from blood cultures requires isolated colonies and generally takes 18 to 24 h after positive signaling on continuously monitored, automated blood culture systems. Rapid non-molecular and molecular methods are currently available to detect the presence of MRSA directly from clinical specimens [29,77]. Today, new technologies are available to rapidly obtain bacterial identification and antibiotic susceptibility results, maintaining high efficiency and sensitivity. Rapid diagnostic procedure represents one of the essential actions needed to improve BSI diagnosis [78]. Rapid detection of MRSA in cases of bacteremia and early notification of results in conjunction with an antimicrobial stewardship program can potentially reduce unnecessary antimicrobial costs and improve care.

## Figures and Tables

**Table 1:** Commercially available blood culture-independent and -dependent methods for rapid detection of *S. aureus* and MRSA for the diagnosis of bloodstream infection.

System (Manufacturer)	Principle	Time to Result (Hours)	Methicillin Resistance Detection ^a^
**Blood Culture-Independent Methods**			
Septifast (Roche Molecular Diagnostics)	Multiplex real-time PCR	4–6	yes
MagicPlex (Seegene)	Multiplex real-time PCR	3–5	yes
SepsiTest (Molzym)	Broad range PCR + sequencing	8–12	no
VYOO (Analytic Jena)	Multiplex PCR + gel electrophoresis	7–8	yes
PLEX-ID Iridica (Abbott Molecular)	Multiplex PCR + ESI-MS	6–8	yes
T2Dx bacteria panel (T2 Biosystems)	PCR + magnetic resonance technology	6	no
**Blood Culture-Dependent Methods**			
Microflex (Bruker Daltonics)	MALDI-TOF	0.2–0.5	no
Vitek MS (bioMérieux)	MALDI-TOF	0.2–0.5	no
S. aureus/CNS PNA-FISH/Quick-FISH (AdvanDx)	FISH with PNA probes	0.5–3	no
Verigene Gram-positive blood culture (BC-GP) assay (Luminex)	Multiplex PCR + solid-microarray detection	2.5	yes
Prove-it Sepsis (Mobidiag)	Broad range PCR + microarray	3.5	yes
GeneXpert MRSA/SA BC Assay (Cepheid)	Multiplex real-time PCR	1	yes
BD Max StaphSR assay (BD)	Multiplex real-time PCR	1.5	yes
FilmArray BCID panel (BioFire; bioMérieux)	FilmArray BCID panel	1	yes
Eazyplex MRSA (Amplex BioSystems)	Loop-mediated isothermal amplification	0.5	yes
Accelerate Pheno System (Accelerate Diagnostics)	FISH (ID) + morpho-kinetic cellular analysis (AST)	1.5 (ID)7 (AST)	yes
Direct Tube Coagulase Test	Clot formation by rabbit plasma	2–24	no
BinaxNOW Staphylococcus aureus (BNSA) (Alere)	Immunochromatographic test	0.5	no

^a^Based on genotype (presence of *mecA*; presence both of *mecA* and *mecC*) or phenotype analysis.

**Table 2:** Rapid antimicrobial susceptibility testing phenotypic methods to detect *S. aureus* methicillin resistance directly from positive blood cultures, bacterial pellet or early (3–4 h of incubation) subculture.

System (Manufacturer)	Principle	Time to Result (Hours)
Accelerate Pheno System (Accelerate Diagnostics)	Morpho-kinetic cellular analysis	7
Phoenix (BD)	Broth microdilution	7
Vitek (bioMérieux)	Broth microdilution	7
Several systems/manufacturers ^a^	Disk diffusion methods	16–24
Etest (bioMérieux), MIC Strip (Liofilchem)	Gradient strips	16–24
Alfred AST system (Alifax)	Turbidimetry	5
Microflex (Bruker Daltonics)	Mass spectrometry-based tests	3–4
PBP2a detection test assay (Alere)	Immunochromatographic test	<1

^a^ Several manufacturers can produce antimicrobial disks for disk diffusion method.
